# Jinx, Control, and the Necessity of Adjustment: Superstitions Among Football Fans

**DOI:** 10.3389/fpsyg.2021.740645

**Published:** 2021-10-06

**Authors:** Orr Levental, Udi Carmi, Assaf Lev

**Affiliations:** ^1^Department of Physical Education and the Research Center for Sports, Tel-Hai Academic College, Upper Galilee, Israel; ^2^Department of Sports Therapy, Ono Academic College, Kiryat Ono, Israel

**Keywords:** superstitions, football (soccer), fandom, behavior, Israel

## Abstract

Superstitions are behaviors human beings use to gain a sense of control over certain events in their lives. Thus, sport and its inherent uncertainty provide fertile ground for superstitious behavior. Research on this subject has focused mainly on athletes while examining the behavioral expressions, motivations, and characteristics of fans’ superstitions that have remained marginal; therefore, the present paper aims to address this lacuna by analyzing these behaviors as part of sports fandom and fans’ daily routines. Semi-structured interviews were carried out with 24 Israeli football fans of local teams. Key findings illustrate two themes: time dependent acts of superstition; and place. It is argued that superstitious behavior serves fans in two ways. First, making them feel their actions influence the match outcomes and helping their favorite team beyond simply cheering in the stands. Second, reducing the level of uncertainty and subsequent stress level characteristic of being a sports fan. Furthermore, following the outbreak of the COVID-19 pandemic when fans’ access to stadiums was restricted, the results show that the fans’ absence from the stadiums led to a reduction in the quantity and frequency of their superstitious behaviors, pointing to the significance of sporting venues in fan behavior.

## Introduction

Calin-Jageman and Caldwell (2014) define “superstitions” as irrational thoughts that connect an object, action or outside source to a particular event and have an impact on the event’s outcome. Keinan (2002) claims that superstitions provide individuals with a sense of control, particularly when experiencing uncertainty and stress. Since competitive sport is rife with such characteristics, professional athletes, coaches, and fans tend to hold a variety of superstitions. Some of these invented superstitions become part of the team legacy or turn into permanent rituals supposedly identified with the team for many years (Dodo et al., 2015). Existing research literature extensively discusses superstitions among athletes (Neil et al., 1981; Flanagan, 2013; Sasvári et al., 2019) yet rarely addresses superstitions among fans. Studies on this topic were mostly quantitative, designed to map such superstitions and identify their characteristics (Kose et al., 2015). This paper aims to provide a qualitative analysis of football fans’ superstitions, while focusing on the motives, prevalence and characteristics of such behavior. Furthermore, the paper discusses the behavioral changes that took place during fans’ absence from the stands due to the COVID-19 outbreak while considering fundamental changes in the sport world during this period as experienced by athletes, referees, and managers (Samuel et al., 2020).

## Literature Review

### Motives for Superstitions

Superstitions have been a basis for decision-making throughout human history. These beliefs have an evolutionary role related to humans’ abilities to explain supposedly threatening events over which they have no control (Rozin and Nemeroff, 2002). Similarly, for sport fans, watching a competition is accompanied by negative emotions and suspense deriving from their uncertainty and limited ability to control the outcome for their team (Knobloch-Westerwick et al., 2009). This lack of control over the outcome of a sport competition may generate psychological and physiological stress among fans. One way to deal with uncertainty is through repetitive behavior before and during an event, such as the behaviors exhibited among professional athletes (McDonald and Karg, 2014). This lack of control, and subsequent repetitive behavior meant to generate control merge into the uncertainty assumption, according to which the outcome of an event is the product of a combination of controlled and uncontrolled forces. Burger and Lynn (2005), for example, found that athletes who believed in the impact of uncontrolled forces on the outcome also believed in the power of superstition and its ability to change the event considerably more than did athletes who felt they had control over the outcome. In this context, the linkage between superstitions and consumerism ought to be highlighted. Vaidyanathan and Aggarwal (2008) note that actively choosing an object and adopting social norms can contribute to superstitious behaviors. In this context, sports fans can purchase clothes or scarves of their favorite team or alternatively adopt certain behaviors because they are common occurrences within sports fandom. For example, in the Block and Kramer (2009) study, the researchers looked into Taiwanese consumer superstitious behavior and demonstrated that local beliefs regarding lucky colors and numbers shape purchase decisions. In another example, the Hamerman and Johar (2013) study on conditioned superstitions contends that sports fans can link two randomly juxtaposed events like drinking a particular beverage brand and winning the game. This linkage creates a conditioned behavior in which the fan will continue to purchase the same brand because he believes it will improve the performance of his favorite team.

Various levels of upsetting events may influence an individual’s perceived control. Several studies found that intense exposure to stress and situational danger was causally related to the adoption of superstitions (Malinowski and Redfield, 1954; Gmelch and Felson, 1980). Another factor that influences the adoption of superstitions and rituals is the need for a high or low sense of control. For example, Keinan (2002) found that level of stress and level of need for control were related to the emergence of superstitious behaviors. Therefore, in the context of highly stressful events we should expect more superstitious behaviors in groups with a high need for control than among groups with a lower need for control. This finding supports the control motivation assumption, according to which stress undermines sense of control while superstitious behaviors can, to some extent, help regain a sense of control. Aside from the need for self-control during stressful events, superstitious behaviors may originate from biases related to active searching for the causes of an event’s outcome (Beck and Forstmeier, 2007). One bias related to superstitions is fundamental attribution error in discovering the cause of an event’s outcome (Zuckerman, 1979). This bias protects fans because it supposedly absolves them from responsibility for failure and gives them credit in case of success (Bradley, 1978).

Quite often the relationship between fans and their team is saturated with superstitions. Studies have shown that the more fans identify with their team, the more likely it is they will cling to superstitions (Brevers et al., 2011). In other words, psychological identification with the team we support increases the range of behaviors deriving from superstitions (Wann et al., 2001; Wann et al., 2013). Since superstitions are intended to provide some certainty about the future, fans tend to adopt these behaviors to increase their perceived ability to help their team, even though their actual impact on match results is negligible (Wann and Goeke, 2018). Similarly, Dwyer et al. (2018b) found a connection between a negative locus of control and higher prevalence of superstitious behaviors. Since superstitions are meant to create a sense of certainty and sometimes solidarity among members of a community, they can be considered to have a positive social impact. One of the advantages of this behavior can be seen in the mood of the fans following the match result. According to Dwyer et al. (2018b), superstitions contribute to improving mood in the case of victory due to the belief that the superstitious behavior contributed to the outcome. In contrast, superstitious behavior does not harm fans’ mood in case of a loss because they attribute the loss to reasons external to their behavior. Nonetheless, due to the irrational context of such behavior, it is sometimes perceived as a negative aspect of the world of sports (Afroozeh and Hadi Pour, 2020).

In the context of sport fans, we must distinguish between superstitious behaviors and ritualistic behaviors. Regarding the former, Bleak and Frederick (1998) define superstitious behavior in sport as: “actions which are repetitive, formal, sequential, distinct from technical performance and which the athletes believe to be powerful in controlling luck or other external factors” (p. 2). Therefore, as Maranise (2013) argues, rituals, and superstitions are not only different in their means and expected outcome, but serve different levels of meaning to individuals. In this regard, McDonald and Karg (2014) found that fans frequently adopt new rituals. Hence the superstitions, which are supposedly the source of these rituals, are also wide and varied. In reference to ritualistic behaviors, Turner defines “ritual” as “the performance of a complex sequence of symbolic acts” (Turner, 1987, p. 75). Thus, rituals mainly relate to the behavior itself and its symbolic function. Similarly, Wann et al. (2001) defined ritualistic behavior as repetitive behavior with symbolic meaning. Therefore, wearing a certain shirt or sitting in the same location in the stands can be interpreted as ritualistic behavior. Yet when fans mention that a location in the stands or wearing a certain article of clothing is intended to recruit lady luck to their side, this connection between an action and an independent outcome turns the behavior into a superstition (Wilson et al., 2013).

### Displays of Superstitious Behaviors

The different motives for superstitious behavior are reflected in a variety of behavioral manifestations. This variety is dependent on acquired cultural aspects as well as local contexts (Mandal, 2018). One of these cultural and social characteristics is that engagement in superstition decreases with each additional year of schooling (Mocan and Pogorelova, 2014). This connection may indicate that in different cultures in which education is not manifested in the same way we should expect a different range of superstitions. In addition, Burger and Lynn (2005) point to differences between individualistic and collectivistic cultures, such that fewer superstitions were found in collectivistic cultures along with less attribution of their impact on individual performance in sport (Burger and Lynn, 2005). Indeed, the cultural characteristics of sport in different branches of sport and among different teams produce different manifestations of superstitious behaviors. One characteristic of superstitions is consistency. When examining baseball, Burger and Lynn found that wearing the same uniform provides a sense of luck, while sitting in the same spot in the dugout gives players a psychological advantage. Their study also points to the importance of timing (i.e., before or during the game) in the context of superstitions, for example by eating the same food before a game or tying one’s shoes at a fixed time during the game (Burger and Lynn, 2005). For example, a study of elite football players in Ghana found that superstitious behaviors found expression mainly in the players’ external appearance and in how they wear their uniforms (Ofori et al., 2012). Another study found that floor gymnasts adopt superstitions related to what they eat before competitions, whereas sprinters saw a connection between luck and the shoes and uniforms they wear (Bleak and Frederick, 1998).

Wann et al. (2013) conducted the most comprehensive study thus far about fan superstitions. The results of their survey show that most fan superstitions are connected to wearing certain articles of clothing (shirts, underwear, and socks), making sounds and spouting off recitations during matches, sitting in a particular seat, closing their eyes, drinking or eating a particular food, and taking planned walks to the bathroom or to purchase food. This finding supports the study by Kelley and Tian (2004), who found that University of Kentucky basketball fans relied on an article of clothing as a factor in the team’s success. The importance fans ascribe to clothing and food (Dwyer et al., 2018a), their belief in curses and bad luck (Wann and Zaichkowsky, 2009) and their tendency not to watch the event (Tamir, 2019) also emerged in additional studies conducted on the topic of fan superstitions and rituals. Kose et al. (2015) noted five superstitious behaviors among Turkish football fans. The first entails behavior that supposedly promotes the future victory of their favorite team, as in a certain article of clothing or object or walking along a predetermined path on their way to the match. The second is the use of a totem (animal, plant, or number) that constitutes a spiritual symbol and is believed to be able to change the outcome of the match. The third includes beliefs to ward off bad luck, though these do not necessarily contribute directly to success. The fourth involves good luck charms and practices from outside the world of sport, among them horseshoes or knocking on wood. The fifth is called “ignoring” and finds expression in ignoring events related to superstitions. In light of these findings, it is not surprising that when the mood of fans is under the influence of superstitions, they become more willing to purchase their favorite team’s merchandise (Hanks et al., 2016). Similarly, Dwyer et al. (2018b) found that fans’ mood is also influenced by the superstitions they carry out, for they believe they can influence the outcome of the competition. Moreover, Wilson et al. (2013) found that superstitious behavior is more prevalent before and during crucial matches and less frequent in matches that have no competitive impact.

### COVID-19, Sport, and Superstitions

Due to the role superstitions play in dealing with uncertainty, we should have expected an increase in the scope of superstitious behavior during the pandemic (Schippers, 2020). Yet this assumption does not necessarily indicate anything about attitudes toward existing superstitions that became impossible to maintain due to restrictions on movement and gathering. According to Imber-Black (2020), COVID-19 did not reduce fans’ belief in superstitions, but rather generated some adaptations to them. Indeed, such behaviors and traditions are part of one’s individual identity and serve a psychological and social purpose. The personal and communal importance of these changes can also be seen in adaptations made in long-standing religious traditions that became necessary in response to the restrictions on gatherings during the COVID-19 era (Frei-Landau, 2020).

In reference to sport, in a current study on the effects of COVID-19, Tamir (2020) examined how the cessation of television broadcasts affected Israeli fans. The study found that these broadcasts serve fans on three levels: the ceremonial level, the social level, and the emotional level. Tamir noted that for fans, sport broadcasts are a source of interest and excitement as well as a motive for social encounters. Thus we can assume that regular visits to sports fields and fan rituals in the stands serve the same purposes and that the impact of not attending the game may be even more intense. In other words, for the fans, not attending games creates symbolic distancing, acceptance of the temporary deprivation and hope to quickly return to the previous activity (Tamir, 2020).

More than its functional role, the sport stadium is important for creating and maintaining in-groups (Tajfel and Turner, 1979) – a communal space for fans whose identity is based on their membership in social groups. It is a place for fans to meet, a symbol of the team, and a venue where fans can implement their supportive rituals (Underwood et al., 2001). Nevertheless, virtual spaces such as online social networks can fulfill some of the fans’ personal and communal needs. Thus, for example, sport fans can consume sport information, maintain contact with others in the community, express their opinions and more (Mastromartino et al., 2020). All these directly contribute to the creation of a supportive identity even though they do not have a direct impact on the team’s athletic achievements. The way in which sport is consumed can change and can even facilitate a great deal of flexibility for those watching from home (Majumdar and Naha, 2020). Yet the nature of this consumption does not necessarily affect fans’ emotional attachment to their team. Furthermore, without attending the matches, fans do not have the opportunity to perform their usual ritual behaviors, such as wearing the team colors, watching the match together with other fans and externalizing their emotions. To meet this need, traditions or rituals must be moved to the home spaces, for example through virtual meetups or by providing the opportunity to purchase the food products available at the stadium by delivery to fans’ homes (Simmons et al., 2020).

The above review of the literature points to three aspects of superstitions that constitute the underlying assumptions of the current study. First, superstitions contribute to individuals’ perceptions of control in the face of uncertainty. Their widespread prevalence in sport emerges from the uncertainty inherent in sport competitions, and particularly in football. Second, superstitions are a cultural expression of the society in which they exist. Therefore, we expect that fan superstitions will be related to the fans’ identity components, such as clothing, speech modes, and emotional attitude toward place. In other words, we hypothesize that superstitions adopted by fans will be a direct continuation of their moral, personal and communal expressions and their interpretation of what it means to be a fan. Third, the COVID-19 pandemic will lead to a change in the nature of the connection between fans and their team and therefore to a change in the behavioral expressions of their superstitions.

## Materials and Methods

This study aims to describe and analyze the behavioral sequence underlying the superstitions of Israeli football fans and the influence of the COVID-19 era on such behavior. Therefore, examining the subjective perceptions of the interviewees—the football fans—is of major importance. Due to the personal nature of these behaviors, we chose the qualitative approach. The chosen study method was a semi-structured interview that included 18 consistent, open-ended questions. As Sparkes and Smith (2013) suggest, semi-structured interviews allow the participants to provide personal context and importance to their feelings and behaviors. The content of the questions referred to personal information and team support in general (e.g., When did you start following your favorite team? How do you describe your fandom? How do your actions as a fan contribute to the team’s performance?). And more specifically, the variety of superstitious behaviors (e.g., do you have a unique behavior as part of your fandom? Have you witnessed superstitious behaviors among fans? Do certain clothes or seat locations have any special meaning?). The wording of the questions was based on the literature review related to superstitions and mainly on the up-to-date study about superstitions among Turkish football fans (Kose et al., 2015). Sample interview questions focused on matters such as *superstitious behavior performed before/during matches, individual vs. shared superstitious behavior, and observations of superstitious behavior practiced by others.*

The study group included 24 fans of various Israeli football teams: Hapoel Be’er Sheva, Hapoel Tel Aviv, Maccabi Tel Aviv, Beitar Jerusalem, Hapoel Haifa, and Hapoel Petach-Tikva. All the interviewees had strong links to their team, as manifested in attending matches at least once every three weeks, purchasing season tickets for home matches, supporting their team for over 15 years, and finally describing themselves as very attached to the team. Our decision to choose fans with a high level of identification stemmed from the fact that fans who are strongly identified with their team are more likely to engage in superstitions (Wann et al., 2013). Of the participants, 22 were young men between the ages of 23 and 34. Two participants were older (one was 67 years old and the other 51). It should be noted that there is a large number of women football fans in Israel. Moreover, more women in recent decades define themselves as fans and attend the stadiums (Ben-Porat, 2009). However, men are still make up the vast majority of fans in the stands. To avoid behavioral differences stemming from social perceptions of gender, we have focused in this paper solely on men participants.

Eighteen of the participants reside in Tel Aviv, four in Haifa, one in Be’er Sheva and one in Moshav Orot. Even though religion came up in some of the interviews, all the interviewees defined themselves as secular.

Initial recruitment of the interviewees was based on the researchers’ personal acquaintance. This prior acquaintance enabled us to conduct the study among fans whose strong identification was already known to the researchers before beginning the interview, thus preventing potential biases due to erroneous self-reporting. The interviewees were recruited by sending each a personal note. Participants gave their consent to be interviewed after being assured that their anonymity would be maintained through the use of pseudonyms. After locating five interviewees, the researchers used the snowball sampling method to locate additional interviewees. This method is especially effective when the population has the attributes of a closed community and the study topic is sensitive and represents a deviation from the norm (Noy, 2008). Although snowball sampling can limit validity as the selection is not random (Cohen and Arieli, 2011), the initial five interviewees were unfamiliar with each other and were fans of different football teams. This condition led to a broad and diverse population of additional interviewees.

Due to restrictions on movement and on face-to-face meetings during 2020 to prevent the spread of the COVID-19 virus, the interviews took place through Zoom video calls. In some cases, more information was added *via* phone calls. The duration of each interview ranged between 45 and 90 min. The use of video calls served two advantages: the first is the ability of each participant to choose his preferred time and location, thus allowing a more relaxed atmosphere and openness. The second allowed the authors to record the interviews for later transcribing and precise analysis.

The interviews were analyzed using thematic qualitative analysis that included an initial reading of all the interviews while extracting categories. In the second phase, the criteria were united into subthemes based on a comparison between the interviewees’ responses and the assignment of relative importance to certain aspects. The identified subthemes included pre-match behaviors, behaviors during and after the match, social influences, the importance of the stadium and the stands, objects, clothing, personal history, and more. The first two phases were conducted simultaneously by two of the researchers. A comparison between the two did not reveal any significant differences in the analysis, so in the third phase, the subthemes were unified into two major themes: time dependent acts of superstition and place. In the first phase we interviewed 18 fans to map their superstitious behaviors. This phase was conducted during the first three months of 2020. An additional round of six interviews was conducted during March 2021 to identify changes in fans’ behavior after being absent from the stadiums as a result of COVID-19 restrictions. The interview procedure was the same in both phases. In the second phase, the authors added specific questions regarding the effect of the COVID-19 pandemic on their behavior (e.g., Did your absence from the stadium change your behavior as a fan? Did you adopt any new behaviors or rituals during this period?).

## Findings and Discussion

### Time Dependent Acts of Superstition

This theme refers to the various times when fans of Israeli football teams express their superstitious behavior. The following paragraphs indicate that superstitious displays before and during a match are more important than those after the match or on other days of the week, when no match takes place.

#### Before the Match

The interviewees defined the period of time before the match as “during the entire day,” from the time they woke up in the morning until the final whistle. This timeframe is characterized by two aspects. One is the fans’ continuous ritual behavior, which they called “getting in the mood” and the other is behavior related to enjoyment and pleasure before the match. The first behavior included a major characteristic common to most of the interviewees: an ongoing behavioral expression that was meant to serve as a ritual of transition into the match itself. The interviewees stated that they want to ready themselves for the match by attaining certain emotions based on repetitive behavior. One such expression is drinking alcohol on match day. According to R: “*Before the match I go to a bar with friends, and we drink vodka almost to the point of passing out*.” Y also noted: “*I make sure to drink beer so that if it hurts, it will hurt less, and if it is a happy event, we will be even happier*.” Alcohol consumption is partially explained by the need to deal with negative emotions and stress deriving from a state of uncertainty and from fans’ limited ability to influence the outcome of the imminent match (Knobloch-Westerwick et al., 2009). Several fans mentioned other ways of getting into the mood for the match. According to T: “*My father, my uncle and I always sit together two-three hours before a home match, play some PlayStation, talk a bit, to boost our morale*.” Y also mentioned: “*I have my usual way, and I stick to it to get into the mood for the match*.” These examples show the fans’ need to create order in the face of their lack of control over the match outcome (McDonald and Karg, 2014). The superstitious behavior observed among most of the interviewees serves two purposes. The first is to provide them a sense of order in the face of uncertainty, and the second is to remove personal responsibility. In other words, superstitious behavior prevents new behavior which, according to the fans’ belief, may create bad luck in the future. This perception connecting previous behavior with a future outcome was found among the interviewees in the context of early enjoyment. Some of the fans stated that match day is a day that should not be enjoyed too much. According to D: “*I cannot have too many good things happen to me before the match*,” while L stated: “*I believe I need to suffer before the match. I prefer to study and not shower*.” This behavior, according to the interviewees, is intended to “safeguard” their enjoyment of positive match results in the future. In other words, they believe there is a balance in the world such that their own happiness will come at the expense of the team’s success. This perspective derives from people’s active attempts to decode the reasons for the outcomes of events (Beck and Forstmeier, 2007), and in this case represents the association between fans’ behavior before matches and collective success.

#### During the Match

Most of the superstitions that shaped fan behavior took place during matches played by their team. For example, according to L: “*During the match you must not take photos or eat*.” G, another interviewee, added: “*I don’t take photos of set plays or scoring opportunities and I continuously shout out ‘ama, ama, ama’ when the other team is close to my team’s goal*.” A’s says: “*During halftime (I) go to urinate and return to the exact same spot*.” Another example is evident from F’s statement: “*Just prior to the start of the game, I rub my tattoo of my team’s logo with my hand, kiss my hand, and raise it to god. There is no way I will ever forget to do this, it is completely ingrained in my habits. This is what brings luck*.” It is evident that the superstitions before and during the match are similar. Both include behaviors related to place (walking along a certain path or a specific location on the field) as well as specific actions (drinking alcohol or repeating certain words). In addition, both include avoiding certain actions (enjoyment or taking photos) for fear of potential consequences.

Some behaviors during the match are characterized by a direct connection between momentary spontaneous behavior on the part of fans and their team’s success or lack of it. Unlike the pre-match rituals, the illusion of a direct connection between fan behavior and the outcome of the event is not accompanied by a time delay. Fans can find out immediately whether their behavior influenced the outcome. One of the biases associated with superstitions is attribution error, in which individuals try to discover the reasons for an event’s outcome (Zuckerman, 1979). This bias is liable to exert a differential influence on the prevalence and form of behavior before the match compared to during the match, with respect to differences in the immediacy of feedback the fan receives. Therefore, the interviewees saw the pre-game behavior as a ritual that must be performed in that they view it as a personal tradition. They associate this with their habits and not necessarily with their desire to directly influence a certain play. Since among other reasons the need for superstition originates from stress based on emotions of uncertainty and lack of control, when such emotions increase, so does the prevalence of the superstitious behavior. The stress derives from what takes place during the match and from the outcome. What happens during the match is perceived more as dependent on luck than as the result of preplanning. Therefore, the range and scope of superstitions during the match were greater than of the pre-match behavioral rituals. This prevalence corresponds with earlier findings showing that intense exposure to stress and situational danger is related to a higher prevalence of superstitious behavior (Malinowski and Redfield, 1954; Gmelch and Felson, 1980).

#### Encounter With Reality

Thus far, the findings about superstitions and the way they affect fans before and during the match correspond with earlier findings (McDonald and Karg, 2014). Nevertheless, the outcome of the match and what occurs during the match, including disappointments, provide fans immediate feedback for their superstitious behavior. For the most part, the participants explained this dissonance by attributing it to a flaw in their earlier behavior or to simultaneous factors that prevented success. For example, T noted: “*I examine other things that we (group of friends that attend games together) did and think maybe we did not do them well enough*.” D added: “*I understand it is a shitty day*… *it has to be something I did not do right; I mean it’s Maccabi – we don’t know how to not win*”.

This perception held by the fans leads them to reexamine the causes for failure. Yet the interviewees agreed that their superstitious behavior must continue. Some suggest doing so without making any changes, as reiterated by S: “*If we lost it does not change the superstition. I will not stop wearing the underwear or change my usual parking spot*.” C stated: “*I can’t blame what we did, I continued my habits even though we lost, I blame the loss on other things*.” Another approach is to adjust their behavior or replace it with another one, as mentioned by A: “*Sometimes it makes me feel uncomfortable so then I change my lucky shirt*”; Y: “*To me this means that if the team loses, I will never wear this shirt again*”.

These varied responses among the interviewees can point to individual differences in superstitious behaviors, to differences in the level of their connection to customs and superstitions, as well as to the intensity of the perceived attribution between behavior and outcome. Moreover, fans who do not change their superstitions behavior after negative outcomes may be influenced by the self-serving bias (bias in favor of the self). This bias protects fans by distancing them from responsibility for failure and attributing to themselves more responsibility for success (Bradley, 1978). Fans who change their behavior because of a negative outcome clearly attribute the outcome to their behavior, thus meeting the definition of superstitious behavior. Furthermore, it is possible to observe how fans take responsibility for their “irresponsible behavior” and thus are liable to criticize themselves and as a result increase their control over team performance in the game.

### Place

The second theme emerging from the analysis refers to the importance of location for the fans. The term “place” relates to spaces that are significant for fans and are related to their symbolic home—the team’s home stadium. The interviewees mentioned two aspects in this context: their own private homes as comparable to the team’s home stadium and the place where they sit in the stands.

#### Home

In 1628, English barrister Edward Coke coined the phrase: “A man’s home is his castle.” For fans, home represents a safe place and an environment they can control. Therefore, they need superstitions to reduce their uncertainty (McDonald and Karg, 2014), and many of these superstitions are related to rituals that take place in the home space (Saenko, 2005). Similarly, the interviewees’ statements indicated that many of their superstitions related to the team’s success are first performed in their home environment. P stated: “*I have been performing the same ritual of coffee, cigarette, shower, and wearing the red shirt for 34 years*.” T also refers to the importance of rituals at home: “*We meet at the same house, play Sony PlayStation and talk about the match*.” Y added: “*before the game I open the closet at home and always pick the same shirt, no way I will wear a different shirt*”.

The interviewees emphasize two complementary aspects of their home rituals. First, the private home symbolizes the starting point of the entire day. Therefore, a series of superstitions related to success in the match later in the day must be launched in the initial environment. H provides additional evidence for this by claiming that after leaving his home he does not return because that might offset the actions he took before he left. The second aspect is the comparison that fans seek to make between their home and the team’s home stadium. Z, for example, stated: “*Before every match I hang the Hapoel Haifa flag outside the living room window of my Tel-Aviv apartment*.” He continues to describe his need to create visual uniformity in his team support, mainly based on the color of clothing, flags and other objects. Home, A claims, is where support for the team begins. In other words, he does not turn into a fan only upon reaching the stands. Rather, he already adopts his fan identify at home. The fact that the fans mentioned they already put on certain articles of clothing such as “lucky underwear” or a “lucky shirt” at home reinforces their devotion to the team’s success. P described this as follows: “*Being a fan at the stadium is half the battle. What is the point of doing all these things only at the last moment? If I don’t already start this process at home, it won’t work*”.

#### Location in the Stadium – “The Block”

All the interviewees in the study attributed great importance to the specific location in the stadium—the permanent seats of fans who hold season tickets. Even in this case the interviewees emphasize the importance of continuity. They believe that choosing their seat is a one-time event and as in the case of team loyalty, they must also display loyalty to the place. H stated: “*When Hapoel Beer-Sheva played its first match at Turner Stadium we went inside two and half hours before and made a thorough search for our new permanent location, which we haven’t changed until today*.” A made a similar statement about his permanent seat: “*This is one of the most critical parts of the match*.” Furthermore, even other fans who sit in the rows close to them (who they do not know beyond belonging to the same fan community) are part of the fixed order: “*Fans in the rows next to us adjust their location or save our seats for us in case something goes wrong in the usual seating arrangement*”.

The perceived importance of superstitions and their impact on the game’s outcome depend on the level of fan identification with the team (Wann et al., 2013). The fans interviewed for this study exhibited high identification with their team and expressed it by continually attending matches for many years. Because the fans make a connection between the outcome of the match and what they do and where they sit, they have continued meticulously observing the rituals they adopted over a long period of time. T states: “*After losses we examine our actions during the match to see whether there are things we need to fix for the next match*.” In the context of the importance of location, A adds: “*When Maccabi was not playing well, we sat in a certain arrangement in the stands and we believed we won the derby because of this seating arrangement. Since then, every week we sit in the exact same spot, which we named ‘the block’ out of the belief that this is what caused the victory. If anyone did not show up to the match it would have destroyed the block’s order and there would have been chaos*”.

The issue of the specific location of every individual fan and of a group of fans together is extremely significant for all fans. Fans believe that seating arrangement and location are connected to the team’s success. Fans who arrive early save these seats “*in case something goes wrong*,” points to the fans’ sense of a shared destiny and also increasing their feelings of solidarity. Nevertheless, not only is location part of a repetitive ritual. It also teaches us about a dimension of sacrifice sometimes manifested in reduced enjoyment from watching the match. A described his experiences at the cup final at Ramat Gan Stadium: “*I chose to sit at the worst place in Ramat Gan Stadium because that is where I sat when we won the cup last year, and even though I could have found a better seat, I chose to sit there again*”.

### Superstitions and Absence From the Playing Fields

In viewing the importance of location in carrying out superstitious behaviors and the prohibition against coming to the field to prevent the spread of the COVID-19 pandemic, this aspect of the study took on new meaning. That is, a fundamental aspect of fans’ identity expressed by going to the matches was taken away from them and they had to find substitutes.

#### Tangible Anchor

When the interviewees were asked to address the issue of the seating and location rituals described above, in most cases they claimed they shifted some of their superstitious behaviors from the stadium to their homes. The interviewees even stated that where they sat in the living room in front of the television was an important attribute of their viewing experience. Indeed, watching matches together and reproducing their ritual behavior (mostly clothing) in the home environment do meet the requirements of time and behavioral expressions. Yet these are irrelevant when the place itself is the anchor of the superstition. In this regard, R stated: “*You do what you can. But I can’t sit in my spot. I can pray anywhere, but prayer has a different meaning in the synagogue*.” Interviewee A added: “*The real force is not me, it is everyone. It’s not me, it’s the place. Our home can help the players even without the fans, and the fans will help even if I am not there, but when all this happens together it is different*.” These statements and others indicate that the fans charge the place with a symbolic and sometimes metaphysical meaning that can be found in the context of sport stadiums worldwide, including in Israel (Levental, 2020). Most interviewees related to the home stadium as the place that allows superstitions to succeed. The place is what connects between the individual’s behavior and the superstition’s desired outcome. Therefore, being absent from the tangible place renders the behavior unnecessary.

#### Distancing

As previously claimed, intense exposure to stress is positively correlated with a higher prevalence of superstitious behaviors (Malinowski and Redfield, 1954; Gmelch and Felson, 1980). Therefore, during a period when fans are prohibited from coming to the playing fields, they should be expected to attempt to preserve and even increase certain behaviors. In other words, because they do not have the option of sitting in the stands, they must find another behavior that can anchor their sense of personal responsibility vis-à-vis their team. According to Mastromartino et al. (2020), under the existing movement restrictions, fans adopted other behaviors that served their personal and communal need to support their team. For example, H stated: “*I found myself constantly following the WhatsApp fan group. It is not a superstition, but it is something that became a routine for me. I felt that if I did not go into the group to be updated, I would be lost*”.

The interviewees’ statements indicate that when they were unable to be present at the playing fields, they replaced their superstitions with rituals. That is, they created new behavioral patterns related to the match, not so much to generate certainty or to influence the outcome, but rather as compensation for their physical distance from the stadiums. G described this well: “*It took me a while but I think I found a new viewing routine. It is something that provides order for me. Yet I have no illusions. Whether or not the team does well has nothing to do with me*”.

In their statements, the interviewees addressed two aspects that helped reduce the quantity and prevalence of superstitions. The first is their reduced ability to influence, which is connected to the physical distance and the absence of any tangible connection as provided by the stadium, the stands or the specific seat. The second is the relinquishing of responsibility. Since part of fans’ identity is their perceived contribution to the team’s accomplishments (Anderson et al., 2012), their absence from the stands relieves them of the responsibility to continue performing certain behaviors they believe are connected to the match outcome.

## Conclusion

While the topic of superstitions has been researched in the context of the general population and even among athletes, only a small number of studies have examined this topic in the context of fans (Wann and Zaichkowsky, 2009; Wann et al., 2013; Wilson et al., 2013; Kose et al., 2015; Hanks et al., 2016; Wann and Goeke, 2018). These studies adopted a quantitative approach and focused on mapping the prevalence of superstitions among fans, the characteristics of their behavior and the potential implications for management. The current study aimed to fill two theoretical voids in the literature. The first entailed cultural differences in the adoption of superstitions, as examined by focusing on Israeli football fans. The second void was the lack of interpretive qualitative research about the significance of these superstitions as part of the identity of football fans. The study also took advantage of the special circumstances brought on by the COVID-19 pandemic to examine how being distanced from the stands led to a change in fans’ identity and consequently in their behavior.

With respect to place, the study found that fans impart meaning to their private homes and to their collective home—the stadium. A comparison is often made between football and religion (Xifra, 2008), with the stadium parallel to the fans’ place of worship. The stadium is the place where fans perform their individual and communal rituals. Nevertheless, the current study found that many rituals are distanced from the sport arena both in space and in time and are performed in the fans’ private homes. Performing these ceremonies at home is also a direct expression of the importance of time. The study participants stated that their rituals began in the morning hours of a match day. In other words, they view the sport competition as an ongoing event that unfolds over more than just 90 min. Since they hold superstitious beliefs that their behavior has an impact on the outcome, they chose to start that behavior as early as possible. An additional component must also be added: their expression of loyalty to the team. They express this loyalty through behavior that is not dependent on a social context or that does not have to be performed in the stands (cheering, singing, waving flags, clothing, and so forth). In this regard, they perceive their individual behavior carried out in the private space as an authentic expression of their sacrifice for the team. Nevertheless, based on the findings of the study regarding fan behavior during the COVID-19 era, this behavior is accompanied or supported by the behavior in the stands and does not stand on its own. Superstitious behavior at home or before the game takes on added value as preparation for the main activity at the stadium and represents an earlier phase of such activity.

Regarding fan behavior during COVID-19, the study findings show that being away from the stands led to a reduction in fans’ emotional involvement with the team and as a result a decrease in their perceived personal responsibility for the team’s outcomes. Since superstitions are meant to provide a sense of certainty on the one hand and a sense of responsibility on the other, the physical distancing was manifested in a reduction in the quantity of superstitions, which were sometimes replaced by rituals. The distancing also led to a reduction in fans’ devotion in carrying out the superstitions.

In conclusion, superstitious behavior serves fans in two ways. One is that such behavior makes them feel their actions influence the match outcomes and therefore can help their favorite team, beyond just cheering in the stands. The second is that superstitions reduce the level of uncertainty and therefore the stress level characteristic of being a sport fan. In view of these needs, behavioral manifestations are a product of the influence of external factors. Soon after their support for the team is formulated and their personality is shaped, their behavior becomes fixed, based on their common cultural and social identity and especially on the fans’ self-image of loyalty and sacrifice. This approach corresponds with the existing research literature according to which superstitions are meant to provide a sense of control in the face of uncertainty (McDonald and Karg, 2014). As the current study found, the superstitions of Israeli football fans meet their personal and psychological need for a sense of control on the one hand and their social need to belong to a community on the other.

Note that this article constitutes an initial effort to examine the superstitions of Israeli fans. Therefore, it seeks to emphasize the local importance of adopting a behavior and to point out similarities to and differences from what we know so far from comparable studies outside of Israel. To deepen the theoretical framework, future research should include a more heterogenous population that will make it possible to examine gender and age factors and their impact on superstitions. In addition, we suggest broadening the research to include fans of lower-level leagues and other popular team sports such as basketball and individual sports such as tennis and athletics. Finally, starting in 2021 fans began gradually returning to the stands. It is important to examine whether upon their return fans will resume their earlier behavior patterns or whether the long absence from the stands perhaps will have an irreversible impact on fan identity and behavior.

## Data Availability Statement

The raw data supporting the conclusions of this article will be made available by the authors, without undue reservation.

## Ethics Statement

The studies involving human participants were reviewed and approved by the Tel-Hai Academic College Ethics Committee. The patients/participants provided their written informed consent to participate in this study.

## Author Contributions

All authors listed have made an equally substantial, direct and intellectual contribution to the work, and approved it for publication.

## Conflict of Interest

The authors declare that the research was conducted in the absence of any commercial or financial relationships that could be construed as a potential conflict of interest.

## Publisher’s Note

All claims expressed in this article are solely those of the authors and do not necessarily represent those of their affiliated organizations, or those of the publisher, the editors and the reviewers. Any product that may be evaluated in this article, or claim that may be made by its manufacturer, is not guaranteed or endorsed by the publisher.
